# Investigating the prevalence of body dysmorphic disorder among Jordanian adults with dermatologic and cosmetic concerns: a case–control study

**DOI:** 10.1038/s41598-024-56315-8

**Published:** 2024-03-12

**Authors:** Rand Murshidi, Muhammad Hammouri, Abdallah Al-Ani, Razi Kitaneh, Majd Al-Soleiti, Zain Al Ta’ani, Sami Sweis, Zeina Halasa, Eva Fashho, Malak Arafah, Noor Almaani, Mahmoud Abdallat, Faten Al-Dar’awi, Eman Kittaneh, Besan Jaber, Farah Almudallal, Zina Smadi

**Affiliations:** 1 Department of Dermatology, School of Medicine, The University of Jordan, Amman, Jordan; 2 School of Medicine, The University of Jordan, Amman, Jordan; 3 Office of Scientific Affairs and Research, King Hussein Cancer Center, Amman, Jordan; 4 Department of Psychiatry, Yale University School of Medicine, New Haven, CT 06519 USA; 5 Clinical Neuroscience Research Unit, Connecticut Mental Health Center, New Haven, CT 06519 USA; 6 Department of Psychiatry and Psychology, Rochester, MN USA; 7 Department of Neurosurgery, School of Medicine, The University of Jordan, Amman, Jordan

**Keywords:** Body dysmorphic disorder, Dermatology, Cosmetic dermatology, Skin disease, Jordan, Psychology, Medical research, Quality of life

## Abstract

Body Dysmorphic Disorder (BDD) is an underexplored psychiatric condition in Middle Eastern countries, particularly in patients with dermatologic concerns, where alterations in appearance may elevate the risk of BDD. We studied patients at Jordan University Hospital's general dermatology and cosmetic clinics from July to September 2022, comparing them to healthy controls. Patients with dermatologic conditions were evaluated per the International Classification of Diseases (ICD-10) criteria by trained dermatologists. All participants completed the Dysmorphic Concerns Questionnaire (DCQ), Perceived Stress Scale, Patient Health Questionnaire-2, General Anxiety Disorder Assessment tool-2. We assessed BDD prevalence using four DCQ cutoffs: 9, 11, 14, and 17, reporting effect sizes as odds ratios (OR). Our study involved 1500 participants, with an average age of 29.3 (± 14.8) years and a female-to-male ratio of 3.15-to-1. At the 9, 11, 14, and 17 DCQ cutoffs, BDD prevalence was 78.2%, 54.2%, 26.5%, and 11.7%, respectively. Patients with dermatologic concerns were more likely to exhibit clinical BDD symptoms than controls at the 11-cutoff (OR: 1.26; 95% CI 1.01–1.58; p < 0.05). Conversely, those with cosmetic concerns were more prone to clinical BDD than controls at cutoffs 9 (OR: 2.26; 95% CI 1.28–3.97; p < 0.05) and 11 (OR: 1.50; 95% CI 1.03–2.20; p < 0.05). Our logistic regression revealed consistent associations between higher DCQ scores and elevated anxiety, depression, perceived skin disease-related stigma, and reduced quality of life (p < 0.05). In conclusion, patients with dermatologic issues and those seeking cosmetic procedures are at significant risk of developing BDD, necessitating proactive screening and referrals for specialized care by dermatologists due to the associated psychological distress and unproductive consultations. Providing specialized training for healthcare professionals to establish an integrated care approach to address the needs of patients with BDD should be the focus of future research projects.

## Introduction

The skin, which is at the forefront of what is noticeable, is central to an individual’s appearance and body image^1^. Many visible skin diseases are associated with distress related to body image or appearance and are often linked with stigmatization^2,3^.

Affecting around 2% of the general population, body dysmorphic disorder (BDD) is a common psychiatric disorder that is often underdiagnosed^4^. It is described as an extreme preoccupation about a self- perceived defect^5^. These preoccupations often times translate to repetitive behaviors like excessive mirror checking, avoiding social gatherings, and overt concealing of said defects^6^. Although the ‘defect(s)’ may not be apparent or minimally noticeable to other people, feelings of shame, depression, reduced quality of life, and even suicide are often associated with this condition^7–9^. Those features of preoccupation, repetitive behaviors, mental acts, as well as clinically significant impairment of psychosocial functioning, are key diagnostic elements for BDD, according to the fifth iteration of the diagnostic and statistical manual (DSM)^10^.

Given the profound impact of skin appearance on body image and mental health, it is important to consider the incidence of BDD and its correlation with dermatologic diseases. As skin diseases are generally associated with a high psychological burden^11,12^, concurrent change in appearance, alongside the socially imposed beauty standards and the idea of perfect skin^1^, may heavily increase susceptibility to heightened psychological distress in said conditions. Moreover, this change in appearance in dermatologic patients may also contribute to feelings of stigmatization, social anxiety, and increased self-consciousness^2,13,14^. As patients with dermatologic conditions exhibit a higher rate of psychological distress than their healthy counterparts, it is only logical to assume that they are at an increased risk to experience symptoms of BDD compared to the general public.

The published literature on this subject is vast. It is notable, however, that most studies lack healthy controls, and are confined to relatively small sample sizes. Furthermore, historically, body dysmorphia has been long considered to be a phenomenon confined within the Western context^15–18^. However, in recent years, with the increasing reach of social media platforms and the adoption of a digital lifestyle due to COVID-19, the literature has shown that this phenomenon has largely become universal^19–21^. Although perceptions of beauty and attractiveness, which are linked to body image, are arguably shaped by consistent factors related to symmetry and skin texture, studies have shown the role of environmental elements in perceptual adaptation^22^. In today’s era, perception of beauty is much more significant due to the pervasive impact of social media and television in everyday life. The constant exposure to a plethora of images through such media may seem trivial, each image incrementally contributes to shaping a person’s standard of beauty to ultimately forming it to align with the standard of the collective imagery they are exposed to^23^. There have been a few reports addressing this in Arab countries with high usage of social media^24,25^.

As patients with symptoms of BDD are more likely to present to a dermatologist or a plastic surgeon than to a psychiatrist^26,27^, and since BDD has yet to receive attention in Arab contexts, with limited literature addressing it, especially in specific populations, such as those seeking cosmetic care or patients with dermatologic conditions^28,29^, and due to lack of regular mental health screening protocols in most healthcare settings in Jordan and the limited literature of psychodermatology within the Arab world and culture, this study explores the prevalence of BDD and the psychological factors associated with it in a group of patients attending general and cosmetic dermatology services in Jordan.

## Methodology

### Design and participants

In this case–control study, we studied the prevalence of BDD across a sample of patients attending dermatology clinics for general dermatologic diseases and cosmetic concerns. Patients with general dermatologic conditions were consecutively recruited from the dermatology clinic at the Jordan University Hospital (JUH), the largest tertiary hospital in Amman, Jordan over a 2-month period from July to September 2022. Trained dermatologists at Jordan University Hospital evaluated their diseases according to the International Classification of Diseases criteria (ICD-10). Questions related to disease onset, disease duration, and any current or past flares related to their skin disease were also answered by the patients.

On the other hand, healthy skin controls were recruited from various departments at JUH. Those included patients presenting for non-dermatologic conditions, caregivers of patients, as well as members of the general population visiting JUH for logistic reasons. Healthy controls were interviewed by trained healthcare workers to exclude any previously diagnosed psychiatric disorder or skin disease. Patients attending the hospital’s general cosmetic dermatology clinic were also invited to participate as well as those attending four other private clinics located across different regions of Amman. These four clinics were chosen as they were part of an affiliate program to train JUH dermatology residents on principles and practices of aesthetic dermatology.

### Measurements

#### Sociodemographic variables

Multiple sociodemographic factors were assessed through various self-reported items. Those variable included age (years), sex (male or female), height (in cm), weight (in kg), level of education, marital status (single, married, divorced, or widowed), employment status, and nature of work (e.g., desk/office or field work).

#### Psychological variables

Self-reported questionnaires relating to depression and anxiety were assessed using the validated Arabic versions of both Patient Health Questionnaire 2 (PHQ-2) and the General Anxiety Disorder Assessment 2 (GAD-2)^30^. Both assessments included two items and were scored on a scale from 0 to 3. After summation, a cut-off point of ≥ 3 indicates positive screening for depression or anxiety, respectively. The simplicity and brevity of both instruments alongside their good reliability renders them excellent options to use in a busy, crowded setting like a dermatology clinic while providing appropriate mental health screening for patients^31–33^.

### Stigmatization due to skin disease

Stigmatization was assessed through a 6-item scale related to the perceived stigmatization due to skin disease on a 4-point Likert scale (1 = not, 2 = a little, 3 = strongly, 4 = totally) with these items: ‘Others are not attracted to me due to my skin disease’, ‘I think that others stare at my skin disease’, ‘Others feel uncomfortable touching me due to my skin disease’, ‘Other people think that my skin disease is contagious’, ‘Other people avoid me due to my skin disease’, ‘Other people sometimes make annoying comments about my skin disease’^34^. A validated Arabic version of this scale demonstrated a high internal consistency with a Cronbach α value above 0.70 as well as satisfactory convergent validity^35^.

### The dysmorphic concern questionnaire (DCQ)

The DCQ assesses concerns about own’s appearance. It consists of 7 items rated on a four-point scale ranging from 0 to 3. Total scoring of the items ranges from 0 to 21. Higher values indicate greater dysmorphic concerns, which in turn, correspond to BDD symptoms. Stangier et al. showcased the advantageous discriminant validity of the instrument through its ability to distinguish between three groups of patients with dermatologic conditions: those who have BDD, patients who suffer from deformities related to skin conditions but without BDD, and patients with non-disfiguring skin conditions and no BDD^36^. Various cut-off values were reported for the DCQ ranging from 9 to 17^36–38^. This instrument is commonly used in assessing BDD in Western culture contexts, and it has not been used in the context of the Arab culture. Hence, a cultural adaptation process of forward–backward translation, expert panel revision, and pilot testing has been utilized. The pre-testing of the questionnaire yielded a Cronbach alpha value of 0.85 thus displaying a high internal consistency.

### The perceived stress scale (PSS)

Developed by Cohen et al., the PSS is a 10-item scale that is a generalized, context-free measure of distress^39^. The PSS-10's simplicity and brevity make it a feasible instrument to be added simultaneously with other measures. In a 5-point Likert scale scoring (0 = never, 1 = almost never, 2 = sometimes, 3 = fairly often, 4 = very often), individuals filling the PSS-10 are asked to rate how much they believe their recent life circumstances were unpredictable, unmanageable, stressful, and uncontrollable. After summation, a total score is calculated with reverse coding of items 4, 5, 7, and 8 since they are positively worded. Higher scores correlate with higher levels of perceived stress. The Arabic version is found on the official website of the instrument and validation of the instrument has shown an acceptable internal consistency with a Cronbach alpha of ≥ 0.70^40,41^.

### Ethical considerations

An informed consent was obtained from all participants prior to filling the questionnaires. The consent form included the participants’ right to anonymity, confidentially of their data, right to leave the study, and reassurance that their participation is completely voluntary, is not associated with any kind of short-term benefit or rewards and does not affect the quality of their received care (if applicable). The authors assert that all procedures contributing to this work comply with the ethical standards of the relevant national and institutional committees on human experimentation and with the Helsinki Declaration of 1975, as revised in 2008. All procedures involving human subjects/patients were approved by the University of Jordan Scientific Committee (ID Number: 2023/598/01).

### Statistical analysis

All collected data were coded, and analyzed using SPSS (IBM Corp, IBM SPSS Statistics for Windows, Version 23.0, Armonk, NY, USA). Categorical variables (e.g., gender) were presented as frequencies [n (%)], while continuous variables (e.g., age) were presented as means ± standard deviations. Categorical associations were evaluated using the Chi-Squared test. Mean differences were evaluated using the t-test and ANOVA tests whenever applicable. Correlations between different psychological and quality of life metrics were evaluated using the Pearson R test. Binary logistic regression was utilized to determine the predictors of BDD at four different DCQ cut-off points (i.e., 9, 11, 14, and 17). Figures were produced using R (version 4.0.2, R Core Team, Vienna, Austria, 2020). A p-value of less than 0.05 was considered statistically significant.

## Results

A total of 1500 participants were included in this study. The study’s sample was comprised of participants with dermatologic conditions (58.7%), cosmetic concerns (9.5%), both dermatologic and cosmetic concerns (1.1%), and controls (30.8%). Female participants comprised 76.0% of the participants. Participants had a mean age of 29.3 ± 14.8 years and a mean BMI of 24.8 ± 5.3 kg/m^2^. The characteristics of participants stratified per group is provided within Table 1.

**Table 1:** Characteristics of included participants.

	Controls	Dermatologic	Cosmetic	p-value
Age	27.85 ± 12.60	30.28 ± 16.41	28.29 ± 10.73	0.217
BMI	24.43 ± 5.15	25.09 ± 5.65	24.06 ± 3.75	0.076
Gender				
Male	81 (17.5%)	268 (30.5%)	8 (5.6%)	< 0.001
Female	381 (82.5%)	610 (69.5%)	134 (94.4%)	
Educational level				
Primary	6 (1.3%)	63 (7.2%)	0 (0.0%)	< 0.001
Secondary	54 (11.9%)	251 (28.8%)	13 (9.2%)	
Undergraduate	377 (83.2%)	502 (57.6%)	122 (86.5%)	
Postgraduate	16 (3.5%)	55 (6.3%)	6 (4.3%)	
Monthly income (JOD)*				
< 1000	220 (47.6%)	570 (68.2%)	56 (39.4%)	< 0.001
1000–2000	153 (33.1%)	197 (23.6%)	49 (34.5%)	
> 2000	89 (19.3%)	69 (8.3%)	37 (26.1%)	
Marital status				
Single	334 (72.3%)	572 (66.1%)	108 (76.1%)	0.011
Married	128 (27.7%)	293 (33.9%)	34 (23.9%)	
Financial stress				
Yes	236 (51.1%)	460 (53.1%)	69 (48.6%)	0.548
No	226 (48.6%)	407 (46.9%)	73 (51.4%)	

*1 Jordanian Dinar (JOD) = 1.41 USD.

Mean DCQ score for the entire sample was 11.7 ± 3.9, which was significantly higher for participants with dermatologic (11.8 ± 4.1) or cosmetic concerns (11.9 ± 3.4) than their control counterparts (11.3 ± 3.6) (p-value = 0.037). A diagnosis of BDD was found in 78.2%, 54.2%, 26.5%, and 11.7% of the total sample based on DCQ cutoff scores of 9, 11, 14, and 17, respectively. Compared to controls, participants with dermatologic disease were more likely to report clinically relevant BDD symptoms at DCQ cutoff 11 (OR: 1.26; 95% CI 1.01–1.58) but not 9 (OR: 0.94; 95% CI 0.72–1.24), 14 (OR: 1.29; 95% CI 0.99–1.68), or 17 (OR: 1.37; 95% CI 0.94–1.93). On the other hand, participants with cosmetic concerns were more likely to have clinical BDD symptoms than their control counterparts at DCQ cutoffs 9 (OR: 2.26; 95% CI 1.28–3.97) and 11 (OR: 1.50; 95% CI 1.03–2.20).

**Figure 1:**
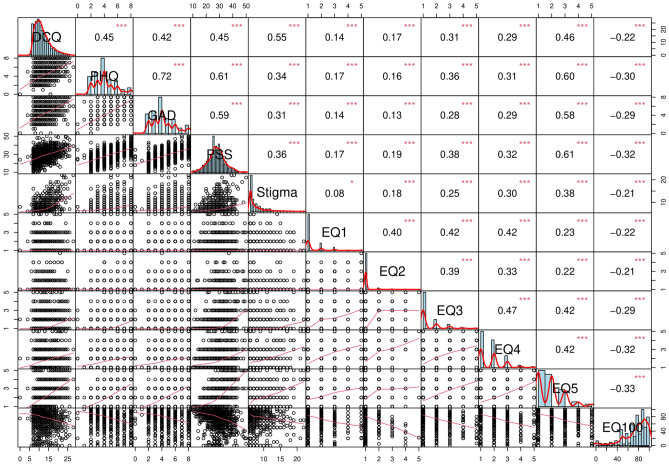
Scatterplot matrix showing the bivariate correlations between DCQ (dysmorphic concern questionnaire) scores and PHQ (depression), GAD (anxiety), PSS (perceived stress scale), perceived stigma scores, and all five EQ components.

DCQ scores correlated positively with PHQ (r = 0.445, p < 0.001), GAD (r = 0.424, p < 0.001), PSS (r = 0.449, p < 0.001), perceived stigma scores (r = 0.543, p < 0.001), and all five EQ components. However, it correlated negatively with the EQ100 score (r = − 0.214, p < 0.001) (Refer to Fig. 1). These correlations remained consistent even when stratified by group.

**Figure 2:**
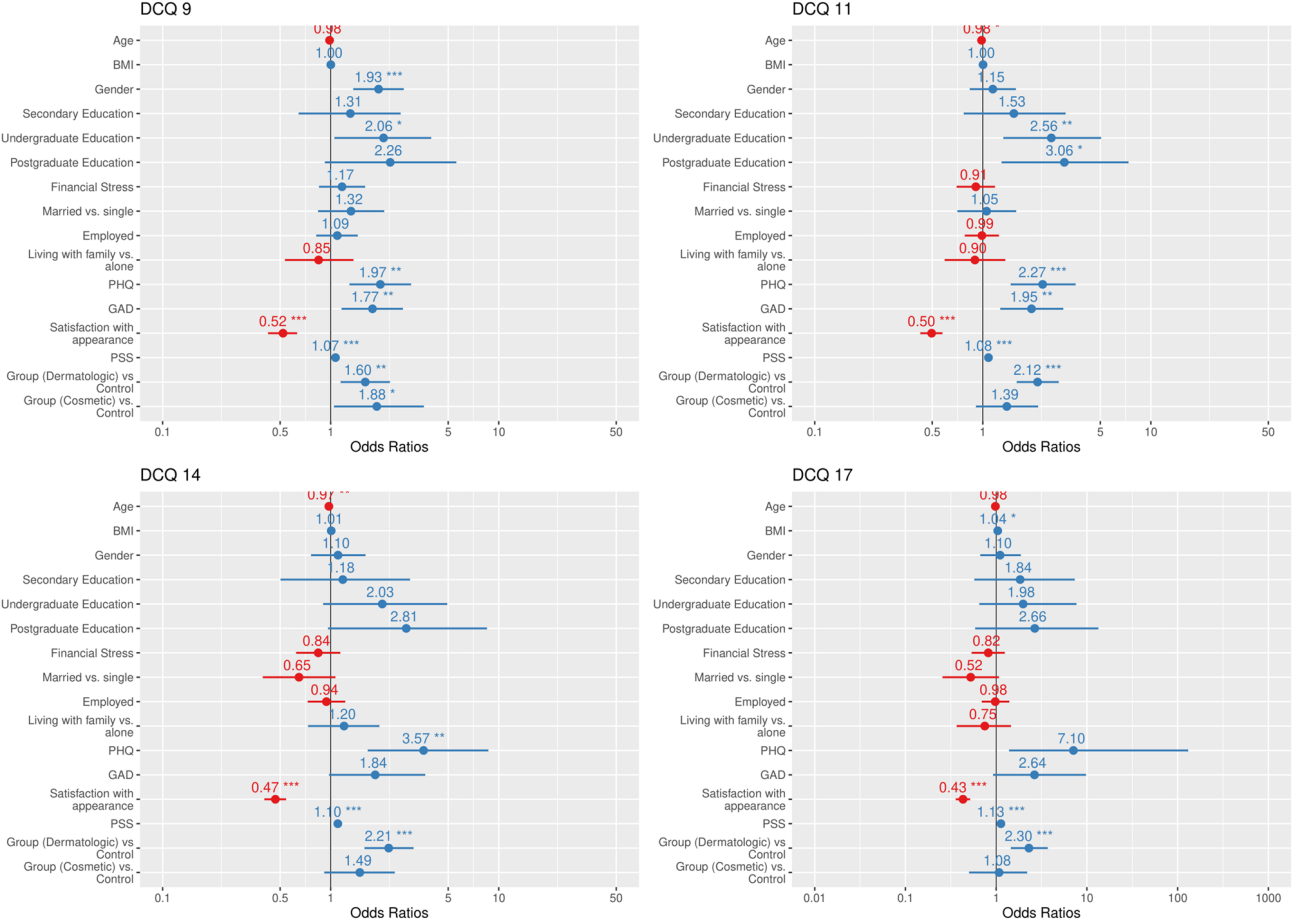
Logistic regression models for predicting BDD at different cutoff values. The odds ratios (OR) and 95% confidence intervals (CI) are shown for each predictor variable. *p < 0.05, **p < 0.001, ***p < 0.0001.

A total of four binary logistic regression models were utilized to find predictors of BDD at the following cutoff values: 9, 11, 14, and 17 (refer to Fig. 2). At a cutoff value of 9, predictors of BDD included gender (OR: 1.93; 95% CI 1.36–2.72), undergraduate education (OR: 2.06; 95% CI 1.07–3.99), PHQ scores indicative of depression (OR: 1.97; 95% CI 1.29–3.01), GAD scores indicative of anxiety (OR: 1.77; 95% CI 1.16–2.70), satisfaction with appearance (OR: 0.520; 95% CI 0.43–0.63), PSS (OR: 1.07; 95% CI 1.04–1.10), cosmetic (OR: 1.88; 95% CI 1.02–3.47) and dermatologic conditions (OR: 1.61; 95% CI 1.15–2.25). Among the aforementioned, satisfaction with appearance was the only negative predictor of BDD.

At a cutoff value of 11, predictors of BDD included age (OR: 0.98; 95% CI 0.97–0.99), undergraduate education (OR: 2.55; 95% CI 1.31–4.98), postgraduate education (OR: 3.06; 95% CI 1.28–7.28), PHQ (OR: 2.27; 95% CI 1.46–3.54), GAD (OR: 1.95; 95% CI 1.27–2.99), satisfaction with appearance (OR: 0.49; 95% CI 0.43–0.58), PSS (OR: 1.08; 95% CI 1.05–1.11), and dermatologic conditions (OR: 1.39; 95% CI 1.59–2.82). Similarly, these factors were predictive of BDD at a cut value of 14 except for level of education and GAD.

Finally, the factors predictive of BDD at a cutoff score of 17 included BMI (OR: 1.04; 95% CI 1.01–1.08), PSS (OR: 1.13; 95% CI 1.09–1.17) and having dermatologic conditions (OR: 2.29; 95% CI 1.44–3.66).

## Discussion

Our study is one of the first to comprehensively assess BDD symptoms in a large sample of patients attending an outpatient clinic for dermatologic conditions and those seeking cosmetic care in comparison to healthy controls. Furthermore, this is the first use of the DCQ instrument to assess BDD in an Arabic speaking population. We estimated the prevalence of BDD using multiple cut-off points of 9, 11, 14, and 17 as published in previous literature, to be 78.2%, 54.2.%, 26.5% and 11.7%, respectively^36–38^. We chose previously mentioned cutoff points due to lack of normative data within the Arabic version of DCQ. Furthermore, these values were derivatives of previously published research on this matter. Stangier and colleagues determined a score of 11 or higher indicates body appearance concerns in females attending a dermatology clinic. On the other hand, a threshold of 14 or more was made to differentiate between individuals with BDD (i.e., those with disfiguring and non-disfiguring disorders)^36^. Other studies determined that a cutoff point of 9 achieves an appropriate balance between sensitivity and specificity^37^. Monzani et al. recommended a cutoff of 17 to differentiate between individuals with BDD and various eating disorders^38^. This study demonstrates that patients with skin disease are at higher risk of developing BDD across all cut-off points. Higher rates of anxiety, depression, perceived stigma related to skin disease, and reduced overall quality of life were almost consistently associated with higher DCQ values across the different cut-offs in our sample. The discrepancies among certain factors, such as GAD not being significantly predictive of BDD at cutoff 14 may reflect the limited psychometric properties of the GAD-2.

Previous studies addressing the same subject have reported BDD prevalence ranging from 1.8% to 57%^29,42–48^. Optimally, diagnosing psychiatric illness is formally done through a structured interview by a trained professional. The use of different instruments to assess BDD across those studies may account for the broad range of prevalence reported. Studies conducted on the basis of a structured clinical interview for the DSM-5 criteria have shown a prevalence of 8.8% in Turkey, 6.7% in Brazil, and 14.4% in the US^17,42,49^. This, however, should not undermine questionnaire-driven data relevant to BDD especially in the context of our study, as it provides significant insight into the phenomenon in dermatologic patients and lays the foundations for future research to further investigate the precedents and antecedents interacting with it. There is significant heterogeneity observed within the published literature on the subject. Sampling techniques, included sample, recruitment settings, how the diagnosis of BDD was made, and the dearth of rigorous large epidemiologic studies would largely explain the variation in prevalence rates^50^.

An interesting finding in our study revealed that stigmatizing perceptions related to skin disease were strong predictors of having BDD symptoms across all cut-off points. This correlates well with a study conducted on a large sample of European dermatologic patients, among which stigmatization was a more relevant predictor of BDD when compared to depression, anxiety, and suicidal ideation^18^. Stigma as a phenomenological construct correlates with BDD as they both share assignment of negative perceptions to a person due to perceived differences from the surrounding population at large. Stigma, however, could arguably be viewed as a larger umbrella encompassing other phenomena as it could occur on basis of mental or physical illness as well and is not confined to appearance, as it is the case for BDD^35,51,52^. The need for educational strategies to reduce stigma relating to skin disease in patients early in their disease course, even before clinically apparent BDD ensues, may benefit these patients^53–55^. Qualitative research delineates that the lived experience of patients diagnosed with BDD in relation to their skin diseases is different than those without any apparent skin conditions and that appearance-related concerns are strongly linked to the experience of stigma^56,57^.

Understanding body dysmorphia or body image concerns should be synonymous with comprehending concepts of beauty and attractiveness. Assumptions that preferences for beauty are acquired through cultural transmission and media exposure have prevailed throughout history. Although true to some extent, cross-cultural and newborn studies have since refuted this and supported the notion that some criteria are established by nature. Humans often regard symmetry, sexual dimorphism, averageness of facial features, and homogenous skin to be attractive^58,59^. These universal standards would explain the dynamic changes in the perceived attractiveness of someone who is affected by skin disease, and how that might initiate feelings of concern towards one’s own body image. With that in mind, as a lot of focus has been given to exploring the intrinsic, almost unconscious tendency to classify a person’s attractiveness, subjectivity is heavily involved when considering individual beauty, especially in the context of self-perception. The concept of self-image is largely influenced and reshaped by societal, cultural, and historical traditions. Understanding these concepts is rather complex and does not follow a rigid formula.

Previous research has highlighted that rates of body dissatisfaction are different between countries of high and low socioeconomic status^60^, different ethnic backgrounds^61^, and even rural and urban settings^62^. The Arab context conveyed in this study is no exception. Numerous research conducted in Saudi Arabia, Lebanon, and Qatar revealed that both women and men perceived their ideal weight as being overweight^63,64^. While a global study revealed that the ideal figure is viewed as slim or even underweight in Western nations like the United States and Germany^65^. Women living in Arab communities may have more concerns regarding their body image as it affects their options regarding marriage^66^. Moreover, values of privacy and conservativeness are heavily emphasized in those communities in comparison to the West. Islamic practices emphasize conservative clothing for women as well. All these factors are important elements in determining the perception of body image and distress related to it.

Our use of multiple cut-off points directs the attention to the important issue of ‘diagnosis threshold’ in psychiatry, and questions whether body dissatisfaction is being drawn to a clinical domain, especially within the framework of cultural differences. This concern is not exclusive to BDD and could be considered in other psychiatric disorders. The argument against medicalizing normal human variation has been made in discussions pertaining normal grief versus major depression, as well as marked shyness versus social anxiety^67–69^.

As we outline the relatively high prevalence of BDD in our sample, and its known associations with other psychological disorders, dermatologists may benefit from training on how to recognize common psychosocial aspects of many dermatologic diseases. This is especially important in BDD, both due to its increased risk in dermatologic patients, and because BDD patients often seek dermatology services rather than psychiatric care. BDD patients often hide their symptoms purposefully, which further delays diagnosis and hinders proper treatment^70^. Moreover, the common stigmatizing attitudes towards mental illness in Arab societies may further contribute to the underdiagnosis of BDD^71^. In addition, the under-recognition of BDD by dermatologists due to lack of formal training or exposure^72^, may render BDD difficult to manage within an Arab context.

We propose some recommendations to remedy the highlighted gaps in managing BDD and other related psychodermatolgic conditions. Our results highlight the importance of recognizing the psychiatric overlap with dermatologic disease, especially BDD. Training both dermatologists and plastic surgeons in the field of psychodermatology and increasing their access to psychiatric services and referrals may be a useful first step as it has been reported that first encounters of BDD patients often occur at those settings rather than a psychiatrist^4^. BDD may be the reason behind a patient’s consultation when there are few objective indicators of skin disease but considerable suffering and numerous complaints. When a high index of suspicion arises for BDD, administering screening questionnaires are warranted^73^. These instruments are fairly short and could be easily incorporated into clinical practice. When appropriate, the patient should be examined for other mental health disorders as well. Professionals in those regards should be also trained to appropriately address those issues in a sensitive manner as constructs of mental health may not be readily accepted in certain populations or age groups^74^. Instruments that can be used to identify and estimate common distress secondary to skin disease, such as the PHQ-2 used in our study, should also be utilized^31,32^. It is important to highlight that the instruments used to screen for BDD in dermatology patients are yet to be validated. As pointed out previously, understanding BDD in a cultural context is crucial. Hence, there should be a standard approach to develop and validate appropriate instruments to accurately screen for BDD and the network of other well-studied psychological variables related to it and establish normative data for the general public or specific groups and settings^75^.

## Limitations

This study has a number of limitations. First, the cross-sectional nature of the study limits the generalizability of the results. Second, the use of self-reported measures and questionnaires might have introduced a number of biases including but not limited to extreme response bias, conformity bias, and recall bias. Inherent biases related to the cultural adaptation process may have also emerged as items of the DCQ questionnaire may not have been culturally sensitive within themselves. Construct bias related to concepts of mental health may have also been a significant contributor to our results. Third, despite having a control group, our sampling frame was limited to individuals presenting to a tertiary university hospital. Fourth, our sample was primarily comprised of young females. Fifth, selection bias. Finally, other factors affecting body image perception were not included, these include suicidal ideation, experiences of abuse, alexithymia, and personality types among others.

## Conclusion

Our study is the first to highlight the understudied phenomenon of BDD in Jordan. We demonstrated that although no specific prevalence of BDD is determined, dermatologic and cosmetic patients are still at a higher risk when compared to healthy controls. Future research should focus on developing and validating culturally appropriated instruments to measure BDD as well as other efficient mental health measures to incorporate in everyday workflow. Recruiting a representative sample composed of wider range of age groups, training dermatologists as well as plastic surgeons and psychiatrists to correctly identify various psychodermatolgic illnesses, and evaluating culturally adapted interventions in BDD patients should be rightly highlighted in future research endeavors on this matter.

## Data Availability

All data/data sets associated with this project can be requested from the corresponding author at a reasonable request.
